# What factors motivate junior doctors to engage as clinical tutors? A qualitative study

**DOI:** 10.5116/ijme.5b07.d108

**Published:** 2018-05-31

**Authors:** Bernhard von Below, Stig Rödjer, Bengt Mattsson, Dominique Hange, Mats Wahlqvist

**Affiliations:** 1 Department of Public Health and Community Medicine/Primary Health Care, The Sahlgrenska Academy at University of Gothenburg, Sweden

**Keywords:** Undergraduate medical education, clinical tutorship, workplace learning, younger doctors, qualitative content analysis, Sweden

## Abstract

**Objectives:**

The study aimed to explore and identify factors motivating junior doctors to engage as long-term clinical tutors in undergraduate medical education.

**Methods:**

In this qualitative study, twenty-seven participants were recruited among junior doctors attending preparatory tutor courses at the Sahlgrenska Academy, University of Gothenburg, and the Primary Healthcare system, West Sweden. They were asked to respond to open-ended questions and write a short account of their needs as clinical tutors for medical students. A qualitative content analysis was performed.

**Results:**

A main theme emerged: “Let me develop my skills in a supportive workplace, provide feedback and merits, and I will continue tutoring”. Participants described suitable personality as fundamental, and the need to develop professional skills, both as clinical tutors and physicians. Tutor education was an important source of knowledge and stimulation. A workplace environment, supporting learning and the tutor’s role, was considered important, including having an adequate time frame. A clear and well-prepared assignment was regarded essential. Junior doctors requested feedback and merits in their work as long-term tutors. Clinical tutorship was considered an optional task.

**Conclusions:**

In this exploratory study, motivating factors of junior doctors’ engagement as future long-term tutors were identified. It is important to form a process where junior doctors can build up professional competence as clinical tutors and physicians. To ensure a sustainable tutorship in the future, we suggest that universities and healthcare authorities acknowledge and further study these motivating factors.

## Introduction

Today, the clinical tutor is a crucial resource in undergraduate medical education.  However, the role of the clinical tutor is in transition, due to recent changes in both education and society. Improvements in clinical education were called for, due to research and development in medical education during the last decades.^1^^-^^5^

Consequently, universities throughout the world have modernized curriculums where medical students begin early developing clinical skills, professional attitudes, and clinical experience.^6^^-^^8^ A clinical tutor has many important roles in a process known as “immersion in practice”.^2^ First, this means that the tutor must be an introducer to the clinical workplace, where students meet patients, relatives, and members of the medical team, consisting of many professions.  Second, the tutor must serve as an instructor of clinical skills, and at the same time, a role model. Third, the tutor needs to be a facilitator of the learning process, showing awareness of core learning goals, and providing necessary room for reflection.^4^ Professional attitudes can then be grounded in the student’s experiences. To orchestrate these advanced tasks, the tutor becomes a coordinator of learning activities in practice.^9^ Thus, tutors arrange clinical situations where students are introduced to early clinical experience, patient contact and communication skills.^6^^-^^8^^,^^10^ Courses with this content help students socially adjust to their profession and interact more confidently with patients.^11^^-^^14^

Over the last years, the importance of a socio-cultural perspective on student learning at the workplace has been discussed.^15^ Research has been carried out to understand student learning processes in workplace learning better.^4^^,^^16^^-^^18^ Thus, the concept of “Community of Practice” was introduced.^19^^,^^20^ It has been defined as a set of relations between persons, activities, and the world, over time, and in relation to other tangential and overlapping communities of practice. A socio-cultural perspective means that what and how medical students learn during clerkship depends on the nature of their interactive experiences, activities, and the meaning that they and others attach to these experiences.^15^   This view leads to the understanding that the clinical tutor plays a central role but is not the only source of student learning in clinical practice. Students learn by participating in the community and culture of the workplace, adequately supported by their tutors. Such learning requires time and continuity.^21^ A shift has taken place from “learning as an acquisition” towards “learning by participation”.^22^^-^^24^

Clinical tutors often face difficulties when combining tutoring with clinical practice.^7^^,^^25^^-^^28^ As an educator, a growing number of students, and lack of time, makes the clinical tutor’s task difficult.^14^^,^^21^ As a clinician, limited economic recourses, subspecialization, managed processes and shorter stays on hospital wards make it even more difficult. When strained by today’s hectic and fragmented health care system, the clinical tutor must prioritize in a multitasking situation.^29^^,^^30^ The task as a clinical student tutor has often been opted out and given lower priority than clinical duties.^28^ These circumstances might result in difficulties recruiting clinical tutors among experienced physicians.

However, recent studies have shown benefits in using junior doctors when introducing students to the clinical environment.^31^^-^^33^ Junior doctors can also be effective teachers and are perceived by students as approachable and empathetic.^34^ As near-peers they might be better placed to understand the needs and situation for medical students at workplace learning.^31^ Earlier research has focused on experienced physicians’ views as clinical tutors.^9^^,^^28^  However, young doctors play an important role in undergraduate clinical education, and studies clarifying their views are rare. Accordingly, we found it necessary to understand junior doctors’ views and motives on serving as future clinical tutors. In this context, the term “junior doctors” implies physicians in training, not yet formally educated as tutors, nor as experienced specialists/consultants.

To reach a deeper understanding of the participants’ views, a qualitative approach was chosen. Qualitative inquiry methods are regarded suitable to attain a deeper understanding from the participant’s perspective.^35^^,^^36^ This study aimed to explore and identify factors that motivate junior doctors to engage as long-term clinical tutors.

## Methods

### Study design and participants

Clinical tutors associated with the Program in Medicine at the Sahlgrenska Academy of Gothenburg University, and the Primary health care system of West Sweden, participate in preparatory tutor courses arranged by the University to qualify for tutorship. Applicants to these courses are junior doctors and have no or little, experience of working as clinical tutors. They may have met students briefly at their clinics, but normally, do not fulfil the requirements for tutorship before attending a preparatory course. The foundation and specialist (residency) programs in medicine include minor tutor courses arranged by the Sahlgrenska Academy and the Primary health care organization. Informants were recruited from participants of these courses.

### Data collection

From January 2014 to August 2015, 478 participants in the tutor mentioned above courses were asked to participate in the study. Seventy-three persons signed an acceptance form. In a letter sent by e-mail, and by regular post, they were then asked to write a short account of their needs as clinical tutors. As an introduction, open-ended questions, similar to an interview situation, were presented: “In your opinion, what factors would motivate or impede you to take on the task of a clinical tutor for medical students? What conditions should be met for you to continue working as a tutor for an extended length of time? Please, share your thoughts and views in writing concerning these issues in your own words consisting of 1-2 pages.”

In the letter, participants were informed that their participation was confidential and that their decision would not affect their forthcoming task as a clinical tutor. Another e-mail with a reminder to respond to the study question was later sent to all those accepting participation. By using a participant recruiting process among a number of tutor courses, a sample with a diversity of age, gender, and experience within our definition of the target group was obtained. Participants were to write their accounts personally, and send them by e-mail as an attached file, or by regular post, to a researcher associated with the Research and Development unit. This researcher removed participants’ names on each text and forwarded the texts to one of the authors (BB), using encrypted e-mails. Information regarding gender, age, and professional level as a physician was attached to each text.

Twenty-seven physicians delivered texts, twenty-six by e-mail and one by regular post. Among participants were eighteen women and nine men. Median age was 30 (26-43) years. Eleven were foundation doctors, twelve were in their medical specialist training period, and four were specialists.

Ethical approval was obtained from the Regional Ethical Review Board of the University of Gothenburg. By using an intermediator and encryption during the e-mail process, confidentiality was assured. No participants reported negative experiences after participation in the study.

### Analysis

Qualitative content analysis analysed participant accounts. This method is often used in qualitative educational research and was found suitable for our research question.^37^

 The analysis included the following steps:

1.        The texts were read for an overview of the content.

2.        Units of meaning were extracted and coded.

3.        Coded units of meaning were condensed into subcategories and categories.

4.        A theme was created based on the latent content of the categories.

5.        Categories and the theme were compared with the original text to ensure their rooting in the material.

All authors read the entire material for an overview. Four of these performed an individual analysis. The analysis was made step-wise as new texts were received from the informants. The last two texts generated no new data, and data saturation was considered reached.  Several meetings were held to discuss the results, and all authors were involved in the formation of subcategories, categories, and theme. Furthermore, feedback on results was gained in several tutor seminars.

Theoretical perspectives used in interpretation were an experience-based, learner-centred perspective,^4^ and learning by participation in a community of practice.^19^^,^^38^ Participant identities were not known to the authors, but age and gender, along with professional level as physicians, was known.

## Results

In the analysis, a theme emerged: “Let me develop my skills in a supportive workplace, provide feedback and merits, and I will continue tutoring”. This theme was based on three categories:  1. Develop my personality towards professional skills as a tutor and physician, 2. Workplace culture supports education, 3. Clear-cut assignment, feedback, and merits (Table 1).

### Develop my personality towards professional skills, as a tutor and physician

Participants expressed that personality was the basis, upon which competence as a tutor could be built. Some were more suited for tutorship than others, as this resident physician said:

“A conducive factor in becoming a tutor, in my opinion, is, above all, the physician’s personality”.

If a clinical tutor did not commit tutoring, results would be poor, as this foundation doctor said:

“A lack of interest in tutoring will lead to poor results. Students can feel a tutor’s lack of interest within three minutes, creating a situation nonconducive to learning”.

Participants found preparatory tutorial education very important, with an ambition to be well prepared and competent for the task:

Most of all I think that training…before tutoring is important; to receive clear instructions concerning my job as a tutor”.

We also found a fear among some participants, that as young doctors early in their careers, they felt they might have had too little clinical experience for tutoring. Thus, they found it important to gain greater professional competence as doctors to adequately fulfil their roles as tutors, as this doctor expressed:

It’s important to feel I have some knowledge of the area they are to be tutored in. I can, of course, provide support even if I’m not as knowledgeable as a consultant, but it feels easier to take on the job of a tutor if I know so much that I can…guide the discussion or plan…if what the student suggests is incorrect.

Participants also reported that continuous education as a tutor was important, both as a source of gaining greater competence, but for stimulation as well, through meeting colleagues:

I need… to get updated on possible changes to the training setup, but also to gain inspiration and make contact with other tutors to exchange experiences and get tips.

### Workplace culture supports education

Many participants reported that the attitude at the workplace towards tutoring and meeting students was very important. They sought a positive educational culture:

Another positive factor to becoming a tutor is to work at a place inspired by education that encourages others to tutor.

And:

An atmosphere at the workplace where other colleagues supervise students and express that tutoring is fun, challenging, and important, is of great importance.

A positive educational atmosphere should include support and acceptance from clinical management, as this foundation doctor said:

It is important that tutoring is seen as something positive from the manager's viewpoint.

This resident, a forthcoming GP, expressed:

Even the management should share the same attitude, considering it a contribution to the training of new physicians, and, furthermore, that it is meritorious for the health center physicians to participate in the learning process, and that it advances one’s own professional development.

Participants also reported that support from colleagues and staff was important and facilitated tutorship:

What close colleagues feel about me supervising students is also important. In most cases, one is dependent on benevolent colleagues when tutoring.

**Table 1:** Codes, subcategories, categories, and theme emerging in the analysis

Codes (examples)	Subcategories	Category	Theme
Personal interest motivational	Personality and skills essential	Develop my personality into professional skills, as a tutor and physician	Let me develop my skills in a supportive workplace, provide feedback and merits, and I will continue tutoring.
Lack of competence as a physician impedes	Education as a tutor and physician, stimulants by colleagues important		
Acceptance and support from management necessary	The positive educational culture at the workplace essential	Workplace culture supports education	
Acceptable workload would encourage	Adequate time for tutoring hinders work overload		
Planning, a necessity	University’s assignment form important	Clear-cut assignment, feedback, and merits	
Positive feedback from students motivational	Feedback, merits, and financial compensation after fulfilled tutorship needed		

Many participants reported the importance of being given adequate time for tutoring students, to avoid too heavy a workload, as this foundation doctor said:

An important factor is, of course, time, and that the workload is not too heavy at your workplace. Best for the students and me is to have time reserved for tutoring.

Adequate time for tutoring would also help informants to fulfil their duties as clinicians, as this resident physician reported:

If I am too busy and feel that tutoring takes time from my clinical responsibilities in such a way that good and safe patient care cannot be guaranteed, it would keep me from accepting tutorship.

Some participants reported that adequate space for students and tutors at the ward or health center when tutoring was also important:

It is important to have adequate space where tutoring is to take place. Too many students in one place can be a burden for patients and staff, bringing difficulties to the role of a good tutor.

### Clear-cut assignment, feedback, and merits

Participants reported the importance of getting a clear and defined task from the university, including information about course plans and study aims. They found it important to have easy ways of contacting the responsible course leaders at the university and sought information regarding the task well in advance. This allowed planning the attachments well, as this resident physician said:

I must have adequate advanced planning from the University so that both myself, as a supervisor, and my manager and scheduler, can plan accordingly. It's about having well-known and established routines so that each tutoring session comes as a natural part of operations.

Some participants wanted to have an influence on course content and form:

A prerequisite for acting as a tutor over a longer period of time is to be able to influence course structure and content.

Participants regarded clear information about the task, its terms and conditions, as important in a process where the doctor could choose to accept or reject the offer to become a tutor. A foundation doctor expressed:

A prerequisite conducive to accepting the task (as a tutor) is first and foremost information on what acceptance implies so that I can decide whether this is something I want to do for myself and my students.

Further, participants emphasized the importance of feedback from students, colleagues, and clinical management on tutoring, as these doctors related:

It's probably very important that students show enthusiasm and interest for me to enjoy being involved in their supervision.

and

I need feedback on my tutoring, not only from students but from colleagues as well, to be stimulated to improve and develop my tutorship.

For some participants, a positive response also included personal financial gain, academic merits, or both:

It is desirable that /tutoring/ is merit considered equally important as research, I believe, not just in the pay envelope but career-wise as well.

and

When tutorial work is well performed it should be rewarded with higher pay.

Participants felt it important for their clinic to be rewarded financially for tutoring students, in order to prioritize education among clinical duties:

Money to the clinic is also needed to motivate receiving students, which puts a strain on the clinic's resources.

## Discussion

Our findings reveal that among this group of young and less experienced physicians, participants identify several motivating factors for continuing their tasks as clinical tutors. These factors concern education, professional competence, and stimulation, as well as the learning culture and organization of the workplace, the importance of adequate time, support, feedback, and merits.

Participants address personality as an important foundation on which competence as a tutor can be built. This is consistent with earlier research, showing that tutors’ non-cognitive characteristics, such as interpersonal skills, and relationship-building, are important when creating a sound learning environment for students.^34^^,^^39^ Research shows that tutor education improves tutoring skills, and can, at least partly, compensate for suboptimal social competence.^40^ Thus, if we look at the patient consultation as parallel to tutoring, research has shown that there may be physicians with less emotional commitment, making it more difficult for them to become engaged in patients^41^  – or in students.  Earlier research has also shown that clinical tutors are often ambitious with high demands on themselves, which can further explain doubtfulness towards the task of a tutor.^28^^,^^42^

Participants express how tutors’ preparatory education is important; they wish to be well prepared and competent. Some participants indicated that they are unsure of their abilities and competence as tutors.Preparatory education can improve a range of competencies necessary to a tutor, including a sense of self, and relationship to others, facilitating growth and development as a tutor.^40^^,^^43^ During preparatory education, tutors can receive information regarding students’ courses, the curriculum of the medical education, and pedagogic methods related to clinical tutoring, such as reflexion and feedback. Continuous tutor education provides opportunities for the enhancement of tutorship, and for discussion and reflection together with colleagues, which participants regard as important. Earlier research shows that such discussions with colleagues are important, but rarely occur in everyday clinical work due to stress and the lack of time.^28^^,^^29^ These occasions are regarded as stimulating by the participants. In Sweden, courses in tutor skills and tutoring are a part of residents’ specialist training, regardless of speciality.  This arrangement has mutual benefits: students learn from younger doctors, but tutoring students is also an opportunity for physicians in specialty training to learn from students. Apart from developing teaching skills they also develop consultation skills when receiving students’ questions, and providing them with feedback on their performance, thus creating a moment of mutual learning. Young doctors are also reinforced when they become aware of how much they have learned.

Several participants describe lack of professional confidence as physicians as a negative factor when tutoring. This is not surprising, as this group of clinical tutors are young, with less clinical experience; some are even foundation doctors. Some participants are worried that there could be some students who know more about specific patient care and treatment methods than themselves, e.g. educated nurses. These concerns can be dealt with during preparatory tutorial education. In an earlier study focusing on experienced tutors, we did not find this fear,^28^ but it is supported by findings in a study, where experienced GP tutors regarded a strong professional identity as a key ability as a clinical tutor.^9^ It should not, however, disqualify younger physicians or foundation doctors as clinical tutors. As mentioned above, findings indicate that junior doctors might be effective tutors, and are often viewed upon by students as being approachable and empathic.^34^ To students, they are easy to identify with and important in developing a sound learning environment, in a community of practice for medical students.

In this exploratory study, participants strongly express the need for a supportive culture of education at the workplace. According to the previously mentioned socio-cultural perspective, and the concept of community of practice, this culture should include the entire staff.^15^^,^^19^^,^^38^ To be able to fulfil the task of a clinical tutor, participants request scheduled time, and support from clinical management and colleagues. They wish to, thus, avoid a multitasking situation, which is quite relevant, as this has been shown to have a negative impact on cognition, health, and professional behaviour.^44^^-^^46^ But mostly, they wish to avoid a conflict of interest with their clinical duties. This is consistent with our findings from an earlier study.^28^ It is of interest to note that compared to the previous study, where participants were experienced clinical tutors, among these younger tutors we see a different attitude concerning adequate time and support. These terms are expressed as a compulsory requirement; if not met they will not accept the task of a clinical tutor and choose not to participate. These participants from a younger generation clearly look upon the task of the tutor as voluntary, negotiating with clinical management about accepting the task or not. In contrast to our earlier research,^28^ this study does not support the view that participants consider tutorship their duty.

Participants voice a wish to get clear information about the task and the course, including course goals from the university, which is more clearly expressed in this study than in our earlier findings.^9^^,^^28^ Of interest is also the request from some participants to influence course content if they are to remain tutors. It might reflect a strong wish from these young doctors to exert greater influence on their working conditions, or a more pedagogic interest among these participants, whereby they are attending tutor education courses.

Participants express the importance of feedback, both from students, their superiors, and colleagues, on tutoring. The literature on feedback principles in medical education is growing rapidly.^47^^-^^50^ Often, feedback to students is discussed in the literature, and is a recurring theme at tutor education courses. However, it is also important for clinical tutors to get feedback, although lack of time and resources at universities and clinics may make this difficult to implement.

Another response on tutoring, which participants find important, is financial compensation and merit gain. This view is clearer than in our earlier findings,^28^ both for the request that tutoring should be an important issue at annual salary negotiations, and for the possibility to gain academic merits and credits. This might also indicate their view of the importance of financial rewards and merits among this younger generation of clinical tutors, which differs from the older generation, at least in Sweden.

We followed guidelines for qualitative research methodology by maintaining open minds and bracketing, i.e., avoiding distortion from our preconceptions during the analysis. ^35^^,^^37^^,^^51^ All authors were physicians, and had been clinical tutors, but had never worked clinically with the participants. They had also been course leaders at the medical faculty and arranged tutor courses, which could potentially influence the interpretation and analysis of data. Consequently, we discussed the importance of awareness of preconceptions thoroughly. We regard that contextual knowledge is essential both when formulating research questions and interpreting results. However, we consider that this knowledge has not distorted our analysis to any significant extent. To strengthen credibility, all authors read the texts several times and participated in the analysis. We held meetings and discussions among the authors to confirm that codes, subcategories and categories appeared logical and consistent. Furthermore, results have been supported at tutorial seminars.

A limitation of this study concerns selection. Participants were recruited among physicians attending preparatory tutor courses arranged in Gothenburg, Sweden, and minor tutor courses during foundation years, and specialty training periods in the region of western Sweden. This would support our aim to recruit physicians with little or no experience of earlier tutorship since they are beginners when they attend a preparatory course. We observed diversity in age, gender, and professional experience in the study. Among those seventy-three who accepted participation, twenty-seven delivered written accounts. Due to confidentiality in the study design, the group that did not deliver texts could not be analysed. However, the reason for this drop-out might have been lack of time, since all were active clinicians, or having more difficulty in formulating their accounts. Lack of interest in the subject was less likely since they had chosen to attend a preparatory tutor course. However, all participants could be regarded as interested in medical education and tutoring. Thus, transferability to other groups of physicians and more experienced clinical tutors may be questionable. Still, we consider the results interesting and relevant to today’s tutors who work in the Swedish health care system, but also in a Nordic or European context, where young colleagues are recruited as clinical tutors.

## Conclusions

In this exploratory study, main motivating factors of junior doctors’ engagement as a future long-term tutor were identified. The study indicates that young doctors have several requests concerning tutorship for medical students. Tutorship is regarded as a voluntary task that can be rejected. Junior doctors want to build up professional skills as physicians and clinical tutors. They also expect active support and merits from their workplaces in order to take on a long-term assignment as a clinical tutor. Clinical tutors play a crucial role in today’s undergraduate medical education, and junior doctors will be needed in this role. To ensure a sustainable tutorship in the future, we suggest that universities and health care authorities acknowledge and further study factors that motivate junior doctors to engage as clinical tutors.

### Acknowledgements

The authors would like to thank the participating physicians who made this study possible. The authors also would like to thank Lena Nordeman, PhD, at the Research and Development unit, Primary Health Care, Southern Älvsborg county, Borås, Sweden, for participation in the process of encrypting the written accounts. Financial support was obtained from the Research and Development unit, Primary Health Care, Southern Älvsborg county, Borås, Sweden.

### Conflict of Interest

The authors declare that they have no conflict of interest.
